# Linagliptin Limits High Glucose Induced Conversion of Latent to Active TGFß through Interaction with CIM6PR and Limits Renal Tubulointerstitial Fibronectin

**DOI:** 10.1371/journal.pone.0141143

**Published:** 2015-10-28

**Authors:** Muralikrishna Gangadharan Komala, Simon Gross, Amgad Zaky, Carol Pollock, Usha Panchapakesan

**Affiliations:** Renal Research Group, Kolling Institute of Medical Research, Royal North Shore Hospital, University of Sydney, St. Leonards, NSW, 2065, Australia; University of Houston, UNITED STATES

## Abstract

**Background:**

In addition to lowering blood glucose in patients with type 2 diabetes mellitus, dipeptidyl peptidase 4 (DPP4) inhibitors have been shown to be antifibrotic. We have previously shown that cation independent mannose-6-phosphate receptor (CIM6PR) facilitates the conversion of latent to active transforming growth factor β1 (GFß1) in renal proximal tubular cells (PTCs) and linagliptin (a DPP4 inhibitor) reduced this conversion with downstream reduction in fibronectin transcription.

**Objective:**

We wanted to demonstrate that linagliptin reduces high glucose induced interaction between membrane bound DPP4 and CIM6PR in vitro and demonstrate reduction in active TGFß mediated downstream effects in a rodent model of type 1 diabetic nephropathy independent of high glycaemic levels.

**Materials and Methods:**

We used human kidney 2 (HK2) cells and endothelial nitric oxide synthase knock out mice to explore the mechanism and antifibrotic potential of linagliptin independent of glucose lowering. Using a proximity ligation assay, we show that CIM6PR and DPP4 interaction was increased by high glucose and reduced by linagliptin and excess mannose-6-phosphate (M6P) confirming that linagliptin is operating through an M6P-dependent mechanism. *In vivo* studies confirmed these TGFß1 pathway related changes and showed reduced fibronectin, phosphorylated smad2 and phosphorylated smad2/3 (pSmad2/3) with an associated trend towards reduction in tubular atrophy, which was independent of glucose lowering. No reduction in albuminuria, glomerulosclerotic index or cortical collagen deposition was observed.

**Conclusion:**

Linagliptin inhibits activation of TGFß1 through a M6P dependent mechanism. However this in isolation is not sufficient to reverse the multifactorial nature of diabetic nephropathy.

## Introduction

The incretin family, including glucagon like peptide 1 (GLP-1), gastrointestinal peptide (GIP) and dipeptidyl peptidase 4 (DPP4), are targets of recent glucose lowering drugs. The DPP4 inhibitors are now well established as hypoglycaemic agents for use in patients with type 2 diabetes mellitus. The potential for DPP4 inhibitors to offer beneficial effects beyond glucose lowering lies with the functional ability of DPP4 to cleave a host of peptides apart from GLP-1.

DPP4 is a serine exopeptidase belonging to the S9B protein family, members of which cleave X-proline dipeptides from the N-terminus of polypeptides, such as chemokines, neuropeptides, and peptide hormones [1]. It is a 110-kDa type 11 integral membrane glycoprotein and is expressed ubiquitously in most organs and cell types. Importantly, DPP4 is therefore able to exert pleiotropic effects. DPP4 exists in both a soluble and membrane bound form, both of which are capable of proteolytic activity. The soluble form in the circulation is thought to arise from shedding of the membrane bound DPP4 and is the target for DPP4 inhibitors as hypoglycaemic agents in clinical use [1]. In contrast, the membrane bound form of DPP4, expressed on the surface of many cell types including kidney tubular cells, endothelial cells and T cells [2], is of major interest with respect to the pleiotropic actions of DPP4. Membrane bound DPP4 also exerts non-enzymatic actions by virtue of co-localising with other membrane proteins and modulating their intrinsic actions [1].

It is widely accepted that transforming growth factor beta 1 (TGFß1) is a major driver of fibrosis in diabetic nephropathy. We have recently reported that linagliptin, a DPP4 inhibitor, reduces high glucose induced active TGFß1 in human kidney proximal tubular cells [3]. This translated to a downstream reduction in phosphorylated Smad2 (pSmad2) and fibronectin transcription and expression. As high glucose induced total secreted TGFß1 was unchanged by linagliptin, we postulated that the mechanism was related to interference with the conversion of latent to active TGFß1. TGFß1 is secreted in a latent form and requires a complex interplay of soluble signalling molecules in the activation process, which releases it from the latency associated peptide (LAP). Once released from the LAP, the unbound TGFß1 can then bind to its receptor to initiate cell signalling via the Smad pathway. Several other molecules such as plasminogen, thrombospondin-1 (TSP-1) and the cation independent mannose-6-phosphate receptor (CIM6PR) [4] participate in this activation process. Among these candidate molecules, we showed that TSP-1 was not the likely target to explain the inhibition of latent to active TGFß1 [3].

The CIM6PR is a membrane protein that binds mannose-6-phosphate containing proteins (like DPP4 and LAP). We have shown in our previous studies that CIM6PR is central to the activation process of TGFß1 in human kidney proximal tubular cells exposed to high glucose [5]. Given the fact that CIM6PR and DPP4 co-localise on the cell membrane [6], we sought to study the interaction between the two in context of high glucose and to delineate the mechanism by which linagliptin reduces active TGFß1. We also extended our studies to include an *in vivo* model of diabetic nephropathy, and importantly compared the treatment group to a control group with matched glucose levels, to evaluate whether linagliptin has antifibrotic effects independent of its glucose lowering properties.

## Materials and Methods

### Cell Culture

HK2 cells, a primary human proximal tubular cell line (American Type Culture Collection), were grown in Keratinocyte Serum Free Media supplemented with bovine pituitary extract 20–30μg/ml and epidermal growth factor 0.1–0.2ng/ml (Gibco, NY, USA) on coverslips and treated with 5mM glucose, 30mM glucose, 30mM glucose plus 1 μM mannose-6 phosphate (M6P) (Santa Cruz) and 30mM glucose plus 30nM linagliptin (generously provided by Boehringer-Ingelheim, Germany) for 48 hours. The IC50 (half maximal inhibitory concentration) of linagliptin is 1nM and the final concentration in our cell culture system was 30nM[3]. Initial experiments were done using increasing concentrations of M6P ranging from 1nM to 1mM. A final concentration of 1μM was chosen. The rationale for adding M6P is to saturate the M6P binding sites on the CIM6PR. If linagliptin reduces the interaction between CIM6PR and DPP4 through a M6P mechanism, then an excess of free M6P in the cell culture system would reduce recognition of the M6P moiety on the DPP4 and hence reduce any CIM6PR: DPP4 interaction.

### Proximity ligation assay

Duolink In situ Fluorescence kit (Sigma Aldrich, St. Louis, MO) was used as per manufacturer’s instructions. This is based on the principle that a pair of oligonucleotide labelled secondary antibody probes generate a signal only when the two probes have bound in close proximity to two primary antibodies attached to proteins that are co-localised [7]. This technique allows direct visualisation of endogenous protein complexes in specific physiological environments. CIM6PR (Novus Biologicals, CO, USA) and DPP4 (Santa Cruz Biotechnology, USA) antibodies were initially optimised for immunofluorescence after cells were fixed using 3.7% paraformaldehyde, blocked with 2% bovine serum albumin and incubated with primary antibodies overnight. Importantly using the same antibodies, we ensured that linagliptin did not alter DPP4 protein expression with immunofluorescence. Cells were then incubated with both primary antibodies overnight, washed, incubated with secondary antibody probes and then subjected to ligation and amplification. Coverslips were mounted on slides using DAPI mounting medium and visualised with a confocal microscope. Images were acquired using Leica TCS SP5 confocal laser scanning microscope with the 63x/1.4NA objective using thick sections and adjusted to ensure that most of the nuclei were in the same Z plane. Resolution (1024x1024 pixels) and parameter settings were standardised for all the images. A minimum of 100 cells per sample was counted. The number of associations (visualised by red dots) was calculated using Image J Analyse Particles function and corrected for the number of cells, which were stained with DAPI. Experiments were done in triplicate and the data was presented as a mean ± standard error. A p value of < 0.05 was considered significant.

### Animal Model

We used endothelial nitric oxide synthase knockout mice (*enos -/-)* as these have been shown to develop significant changes of diabetic nephropathy[8]. We used linagliptin (provided by Boehringer Ingelheim and at the recommended dose of 3mg/kg per day via oral gavage) as the DPP4 inhibitor in our studies. Current “best practice” for renoprotection rests with administration of an agent that blocks the renin-angiotensin-aldosterone (RAAS) system. Hence we included a comparator with Telmisartan (Sigma Aldrich, St. Louis, MO, at 3 mg/kg /day in drinking water). Animal groups were allocated as shown below for the renal studies, which was conducted for 24 weeks post induction of diabetes. Mice were given intraperitoneal injections of streptozotocin (STZ) at a dose of 55 mg/kg/day (Sigma Aldrich, St. Louis, MO) for 5 consecutive days at 7–8 weeks of age. This is the standard low dose STZ protocol validated by the Animal Models of Diabetic Complications Consortium. Blood glucose was tested using a glucometer (Accuchek Nano, Roche Diagnostics) one week after STZ through tail vein blood collection. Diabetes was defined by blood glucose greater than 16 mmol/L after a six-hour daytime fast. Mice with levels below 16 mmol/L were excluded from the study. Fasting blood glucose levels were measured monthly. Long acting insulin (Insulin Glargine, Sanofi Aventis, Australia) was initiated as required from 10 weeks of age and was administered thrice weekly if the blood sugar exceeded 28 mmol/L or if they had lost weight greater than 25% from baseline. The aim was to match glycaemia and to maintain body weight and avoid ketonuria without achieving euglycaemia. Importantly, in all studies the glycaemic control of the diabetic animals was matched to assess specific renal effects of linagliptin independent of glycaemic control. The study was approved the Royal North Shore Hospital Ethics Committee (Protocol number 1203-009A). The Australian Code of Practice for the Care and Use of Animals for Scientific Purposes were followed in this study. Animals were anaesthetised using short inhalational anaesthesia with 2% Isoflurane for minor procedures. Animals were euthanized under 2% Isoflurane anaesthesia using cardiac puncture terminally. The groups were as below:

(i) Non—Diabetic (control): 12 animals
(ii) Non—Diabetic (control) with linagliptin: 8 animals
(iv) Diabetic: 12 animals
(v) Diabetic with linagliptin: 9 animals
(vii) Diabetic with telmisartan: 9 animals

### Measurement of Physiological Parameters

Body weight was assessed monthly. Blood pressure was measured using a non-invasive tail vein cuff method (CODA BP apparatus, Kent Scientific, USA) preterminally.

### Urine Biochemistry

Urine was collected at two different time points (4–6 weeks after initiation of treatment using metabolic cages and terminally using bladder puncture). Urine creatinine was measured using a picric acid method (Creatinine Companion, Exocell Inc., USA). Urine albumin was measured using Elisa (Albuwell, Exocell Inc., USA).

### Kidney Tissue Harvest

The un-perfused left kidney was harvested and snap frozen, after embedding in OCT compound. The right kidney was perfused with phosphate buffered saline (PBS) followed by 4% paraformaldehyde (PFA) and subsequently fixed in 10% neutral buffered formalin for 24–48 hours.

### Histology and Immunohistochemistry

Formalin fixed paraffin embedded (FFPE) kidney sections were stained with Periodic Acid Schiff and Sirius red stain. Assessment of histological change was done in a blinded manner. The Glomerulosclerotic index (GSI) was calculated based on the formula, GSI = [(1 x N1) + (2 x N2) + (3 x N3) + (4 x N4)]/(N0 + N1 + N2 + N3 + N4), where Nx is the number of glomeruli with each given score for each section. Atrophic tubules were defined by dilatation, epithelial shedding and thinning of epithelium. Tubular damage was scored by counting the number of atrophic tubules per 400 tubules at 200x magnification. The degree of interstitial collagen content in Sirius red stained slides were assessed in a blinded manner using Image J by identifying the percentage of interstitial collagen positive region at X 200 magnification in 5 randomly selected regions. Immunohistochemistry for nuclear pSmad 2/3, was done on 4 micron paraffin embedded sections using goat anti-mouse pSmad 2/3 (SC11769-G, Santacruz Biotechnology, USA), after an overnight incubation at a concentration of 1:100, followed by donkey anti goat HRP tagged secondary antibody (Santacruz Biotechnology, USA at a concentration of 1:100). With respect to fibronectin, the primary antibody (Sigma, USA) was used at a dilution of 1:1000 followed by anti rabbit secondary antibody at a dilution of 1:100 (Dako, Australia) The chromogenic reaction was carried out with 3,3′-diaminobenzidine chromogen (Dako, Australia) solution for 10 minutes.

### RNA isolation and RT-PCR Analysis

Total RNA was extracted from kidney tissue using Qiagen RNEasy Mini kit on an automated RNA extraction protocol using Qiacube. cDNA was synthesised using Roche Transcriptor First Strand cDNA synthesis kit (Roche, USA). The real time PCR was done using SYBR green (Bioline, Australia) for fibronectin (forward-CACGGAGGCCACCATTACT and reverse-CTTCAGGGCAATGACGTAGAT) using actin (forward-CAGCTGAGAGGGAAATCGTG and reverse-CGTTGCCAATAGTGATGACC) as the endogenous control. Primers were sourced from Sigma. The RT-PCR was performed on the AB7900 machine (Applied Biosystems, Australia).

### Western Blot analysis

Frozen tissue was homogenized with Quiagen Tissue Ruptur in 1.5 ml of cold 20mM HEPES buffer, pH 7.2, containing 1mM EGTA, 210mM mannitol, 70mM sucrose and centrifuged at 1.500 x g for 5 min at 4°C. Samples were then analysed by SDS gel electrophoresis (Novex, Life technologies, Australia) and electroblotted to Hybond Nitrocellulose membranes (Amersham Pharmacia Biotech, Bucks, UK). Membranes were then probed with pSmad2 (Ser465/467) antibodies (#3101,Cell Signalling Technology, USA) followed by HRP tagged anti rabbit antibody (Cell Signalling Technology, USA). Membranes were stripped and probed for total Smad2 (#5339, Cell Signalling Technology, USA). Proteins were visualized using Luminata Western HRP Substrate (Millipore) in a LAS 4000 image reader (GE Healthcare Life Sciences). Analysis was performed using Image J software (NIH, USA).

### Statistical analysis

Statistical analysis was done using GraphPad Prism 6. Data are expressed as mean ± standard error of mean. A P value < 0.05 was considered statistically significant. Blood sugar profile during the study was measured using two way repeated measures Anova. Anova with Bonferroni’s correction was used for all other statistical analysis.

## Results

### M6P and linagliptin reduced high glucose induced CIM6PR:DPP4 interaction in HK2 cells

**Fig 1:**
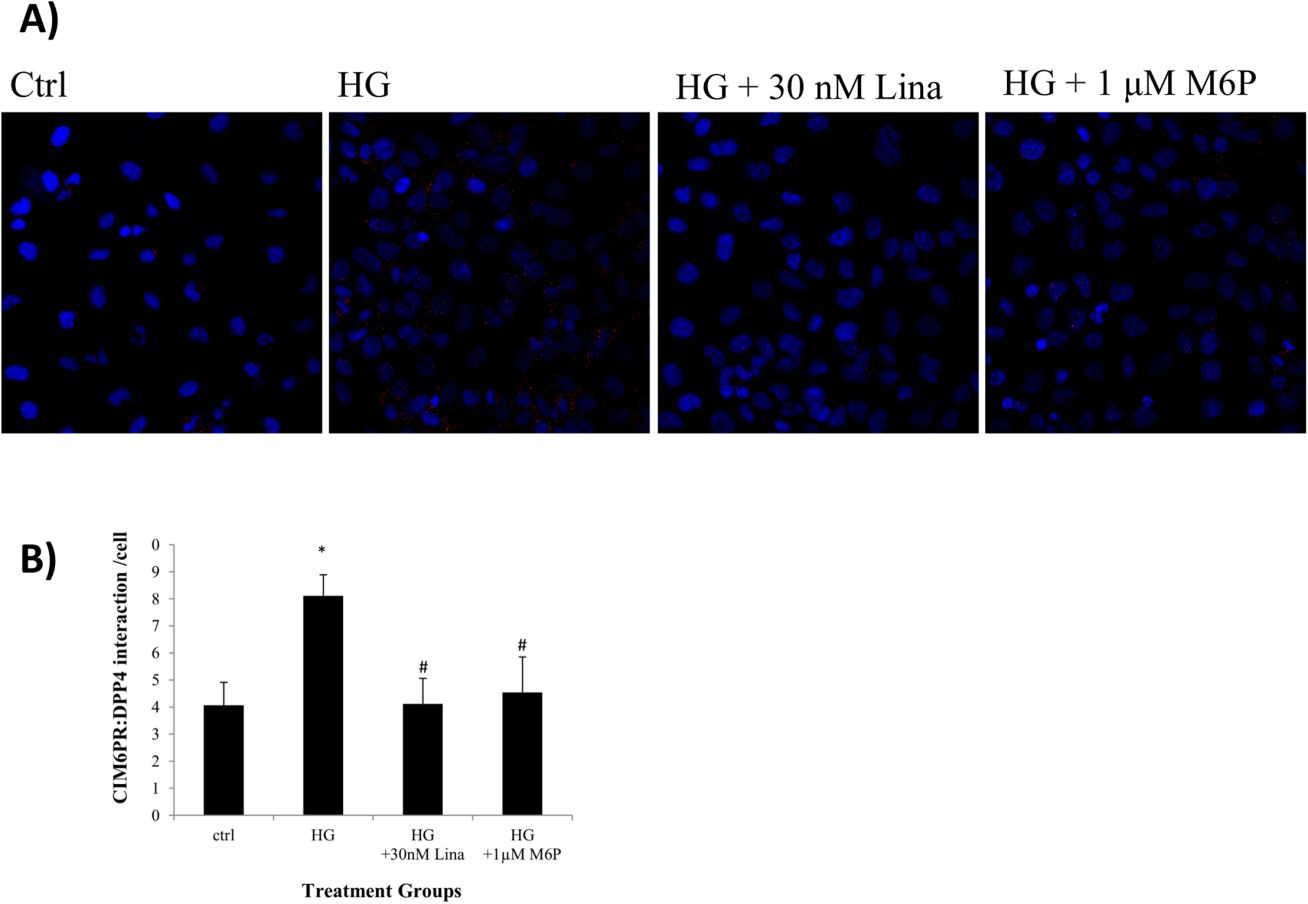
M6P and linagliptin reduced high glucose induced CIM6PR:DPP4 interaction in HK2 cells. **(A)** Proximity ligation assay demonstrating endogenous protein-protein interaction between membranous DPP4 and CIM6PR in HK2 cells visualised as individual fluorescent dots. This is increased in 30mM high glucose (HG) environment compared to control 5mM glucose (ctrl). M6P at 1μM and linagliptin at 30nM reduced the high glucose induced interaction. A quantitation of this is shown in **(B)**. Data is represented as mean ± standard error, n = 3. * = P<0.05 compared to 5mM glucose, # = P<0.05 compared to 30mM glucose.

CIM6PR and DPP4 were both present on the cell membrane of HK2 cells. Proximity ligation assay revealed a significant increase in signal when cells were exposed to 30mM glucose suggesting that CIM6PR and DPP4 were interacting under high glucose conditions (P<0.05). Linagliptin reduced this high glucose induced interaction (P<0.05). Excess M6P was also able to reduce this interaction (P<0.05), which suggests linagliptin is competing with M6P for binding at the CIM6PR (Fig 1A and 1B).

### Blood glucose were elevated and matched in diabetic mice

All diabetic mice had significantly elevated fasting blood glucose compared to control mice (P<0.05). All the diabetic mice had matched fasting blood glucose levels. This is shown in Table 1. This was in the absence of urinary ketonuria (data not shown).

**Table 1:** Metabolic and physical parameters of mice.

	Control	Control + linagliptin	Diabetic	Diabetic + linagliptin	Diabetic + telmisartan
Gain in weight (grams)	5.86 ± 0.70	4.66 ± 0.70	-0.68 ± 0.63**	-0.53 ± 0.60**	2.20 ± 0.64^##^
Left kidney/ body weight ratio (%)	0.84 ± 0.05	0.81 ± 0.03	1.07 ± 0.06**	0.97 ± 0.05	0.83 ± 0.02^##^
Average blood sugar (mmol/L)	9.9 ± 0.1	10.2 ± 0.1	20.9 ± 0.9*	22.4 ± 0.7*	22.4± 1.1*
Systolic BP (mm Hg)	115.6 ± 2.7	115.0 ± 3.2	111.1 ± 4.4	102.9 ± 2.5	101.3 ± 4.9
24 hour urine albumin excretion (μg/day)	463.4 ± 105.7	360.8 ± 80.6	2319 ± 438.3**	2238 ± 226	766.4 ± 152.3^##^

Values are shown as mean ± SEM.

* = P<0.05 vs control

** = P<0.01 vs control

^##^ = P<0.01 vs diabetic

### Linagliptin did not reduce albuminuria in diabetic mice

The diabetic mice showed significant terminal urinary albumin excretion in keeping with diabetic nephropathy compared to control mice (P<0.01). This was not improved by linagliptin. However telmisartan significantly reduced albuminuria (P< 0.01). This is summarised in Table 1.

### Physical parameters

The diabetic mice exhibited the expected weight loss during the experiment in comparison to significant weight gain by control mice (P<0.01). The diabetic mice treated with linagliptin also demonstrated weight loss (P<0.01) and were not significantly different to untreated diabetic mice. However diabetic mice treated with telmisartan gained weight, which was significantly better than diabetic mice (P<0.01). The diabetic mice also showed significant renal hypertrophy compared to control mice (P<0.01). Linagliptin did not alter this significantly. Telmisartan reduced diabetes induced renal hypertrophy as signified by the normalised kidney/body weight ratio (P<0.01). The control and diabetic mice had comparable systolic blood pressure. These results are summarised in Table 1.

### Linagliptin did not reduce glomerulosclerosis in diabetic mice

**Fig 2:**
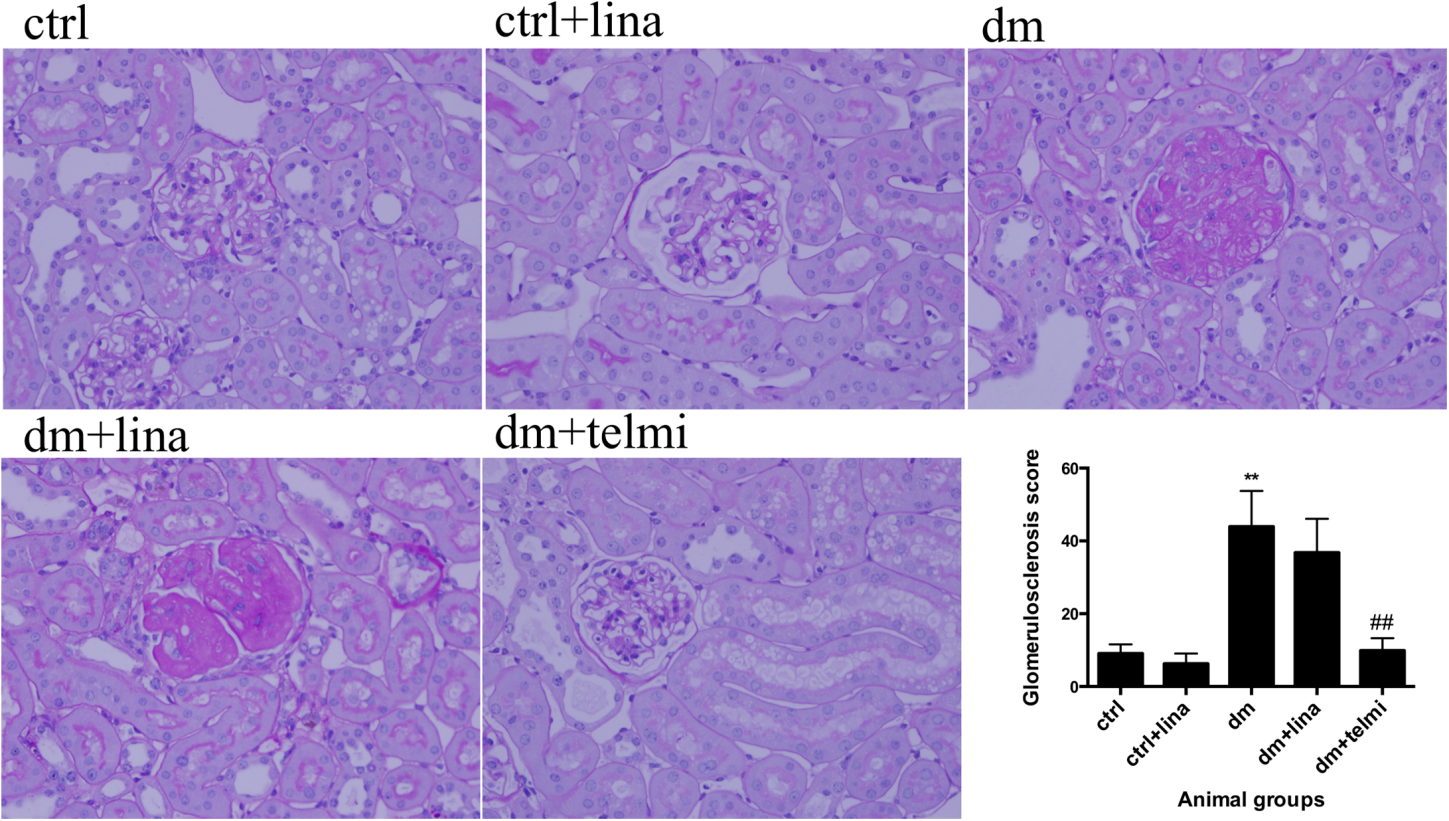
Linagliptin did not reduce glomerulosclerosis in diabetic mice. Diabetic mice demonstrated increased glomerulosclerosis, which was improved by telmisartan but not by linagliptin as demonstrated by quantification of glomerulosclerosis by glomerulosclerotic index. Representative photographs of PAS stained sections for control (ctrl), control + linagliptin (ctrl + lina), diabetic (dm), diabetic + linagliptin (dm + lina) and diabetic + telmisartan (dm + tel) groups are shown (Magnification = original X400). Data are expressed as mean ± SEM with ** = P<0.01 vs ctrl, ## = P<0.01 vs dm.

The diabetic mice had significant glomerulosclerosis compared to control mice (P<0.01). Linagliptin did not ameliorate the degree of glomerulosclerosis in diabetic mice. Telmisartan showed a significant improvement in the diabetic animals (P<0.01) (Fig 2).

### Linagliptin partially reduced tubular atrophy in diabetic mice

**Fig 3:**
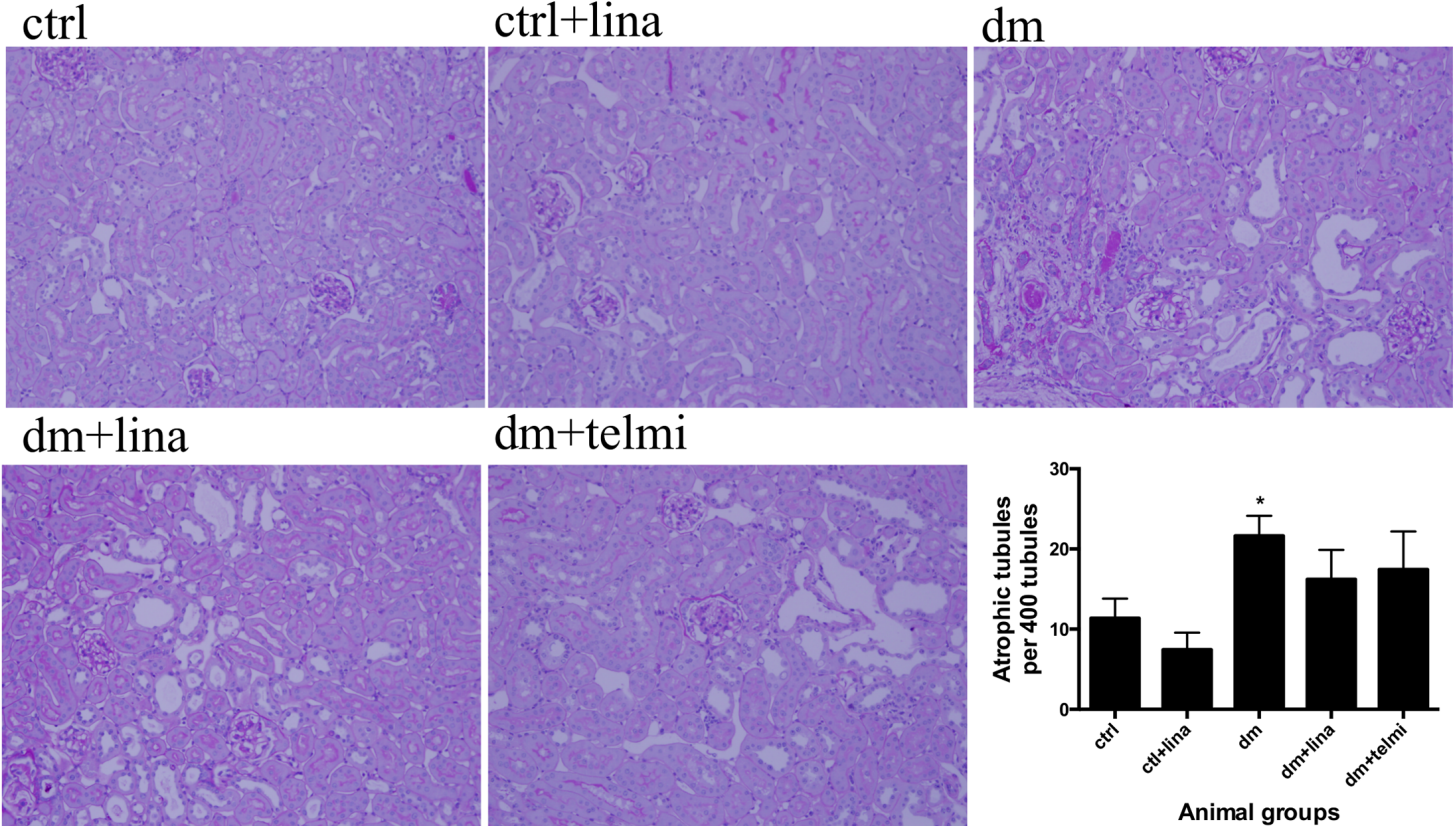
Linagliptin partially reduced tubular atrophy in diabetic mice. Diabetic mice demonstrated tubular atrophy, which was partially reduced by linagliptin and telmisartan. Representative photographs of PAS stained sections of tubulointerstitium for control (ctrl), control + linagliptin (ctrl + lina), diabetic (dm), diabetic + linagliptin (dm + lina) and diabetic + telmisartan (dm + tel) groups are shown (Magnification = original X 200). Quantification of tubular atrophy in all groups was done by counting the number of atrophic tubules per 400 tubule count (Data are expressed as mean ± SEM with * = P<0.05 vs ctrl).

Diabetic mice had significant tubular atrophy (P<0.05). Although both linagliptin and telmisartan showed a reduction in diabetic tubular atrophy, this change did not reach statistical significance (Fig 3).

### Linagliptin reduced tubular pSmad 2/3 expression (a marker of transforming growth factor beta activation) in diabetic mice

**Fig 4:**
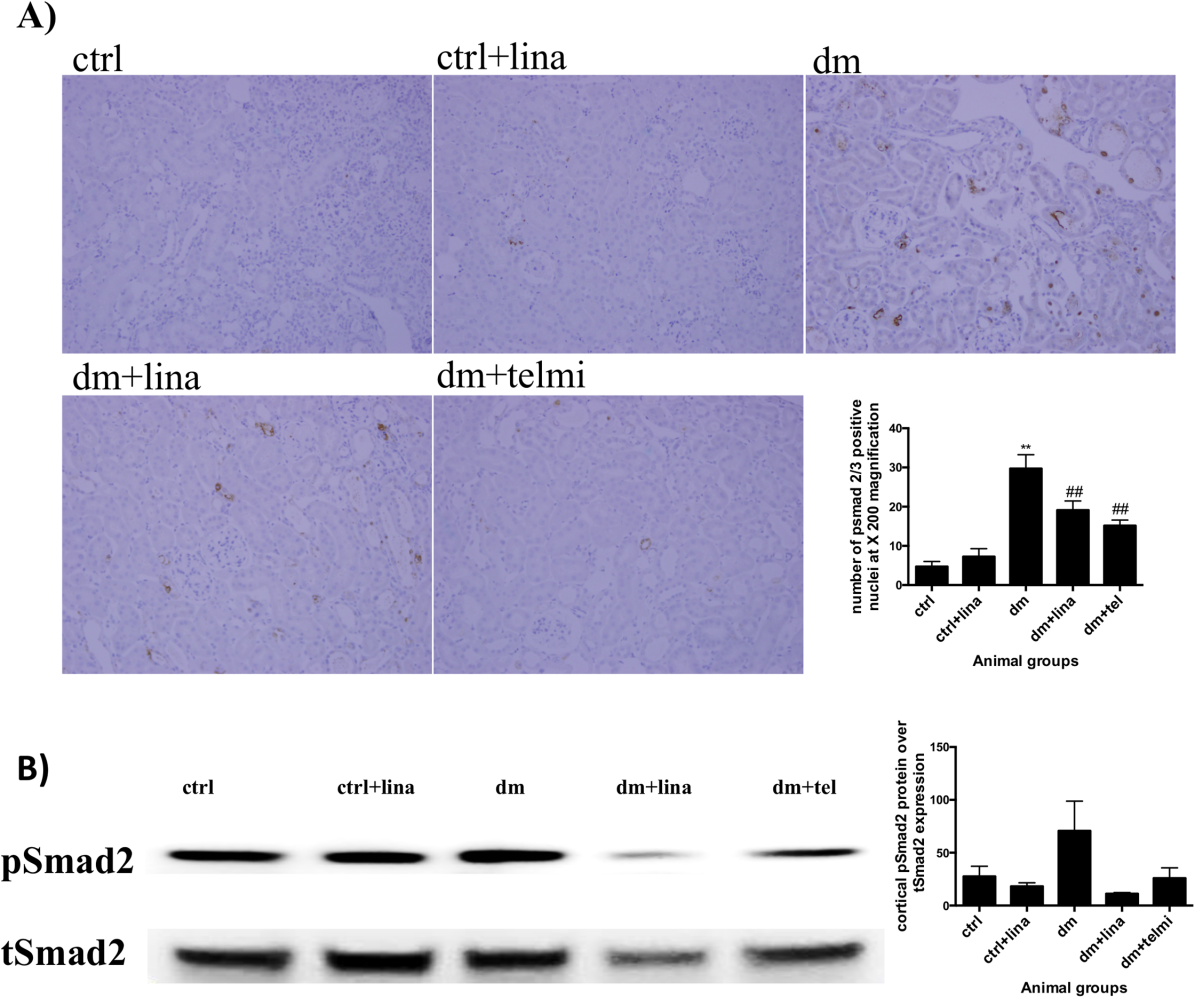
Linagliptin reduced tubular pSmad 2/3 expression (a marker of transforming growth factor beta activation) in diabetic mice and showed a trend towards reduced cortical pSmad 2 expression on western blot analysis in diabetic mice. A) Diabetic mice demonstrated increased pSmad2/3 nuclear expression with immunohistochemistry, which was reduced by linagliptin and telmisartan. Representative photographs for control (ctrl), control + linagliptin (ctrl + lina), diabetic (dm), diabetic + linagliptin (dm + lina) and diabetic + telmisartan (dm + tel) groups are shown (Magnification = original X 200). Quantification was done by counting the number of positive nuclei at X200 magnification. Data are expressed as mean ± SEM with ** = P<0.01 vs ctrl, ## = P<0.01 vs dm. B) Diabetic mice showed a trend towards increase in pSmad 2/total smad2 expression compared to control mice and a reduction with both linagliptin and telmisartan. This trend was consistent with the immunohistochemistry findings but did not reach statistical significance. Quantification was done using Image J. Data are expressed as mean ± SEM, n = 6.

Diabetic mice showed significant renal nuclear expression of pSmad 2/3 (P<0.01), signifying TGFß signalling. Both linagliptin and telmisartan treated mice showed a significant reduction in pSmad2/3 expression (both P<0.01) (Fig 4A). Western blot analysis of pSmad 2 expression showed a trend towards increased expression in diabetic mice, which was ameliorated to some extent by linagliptin (P = 0.08)(Fig 4B). The results from the Western blot analysis were consistent with the findings from immunohistochemistry.

### Linagliptin reduced fibronectin transcription and expression in diabetic mice

**Fig 5:**
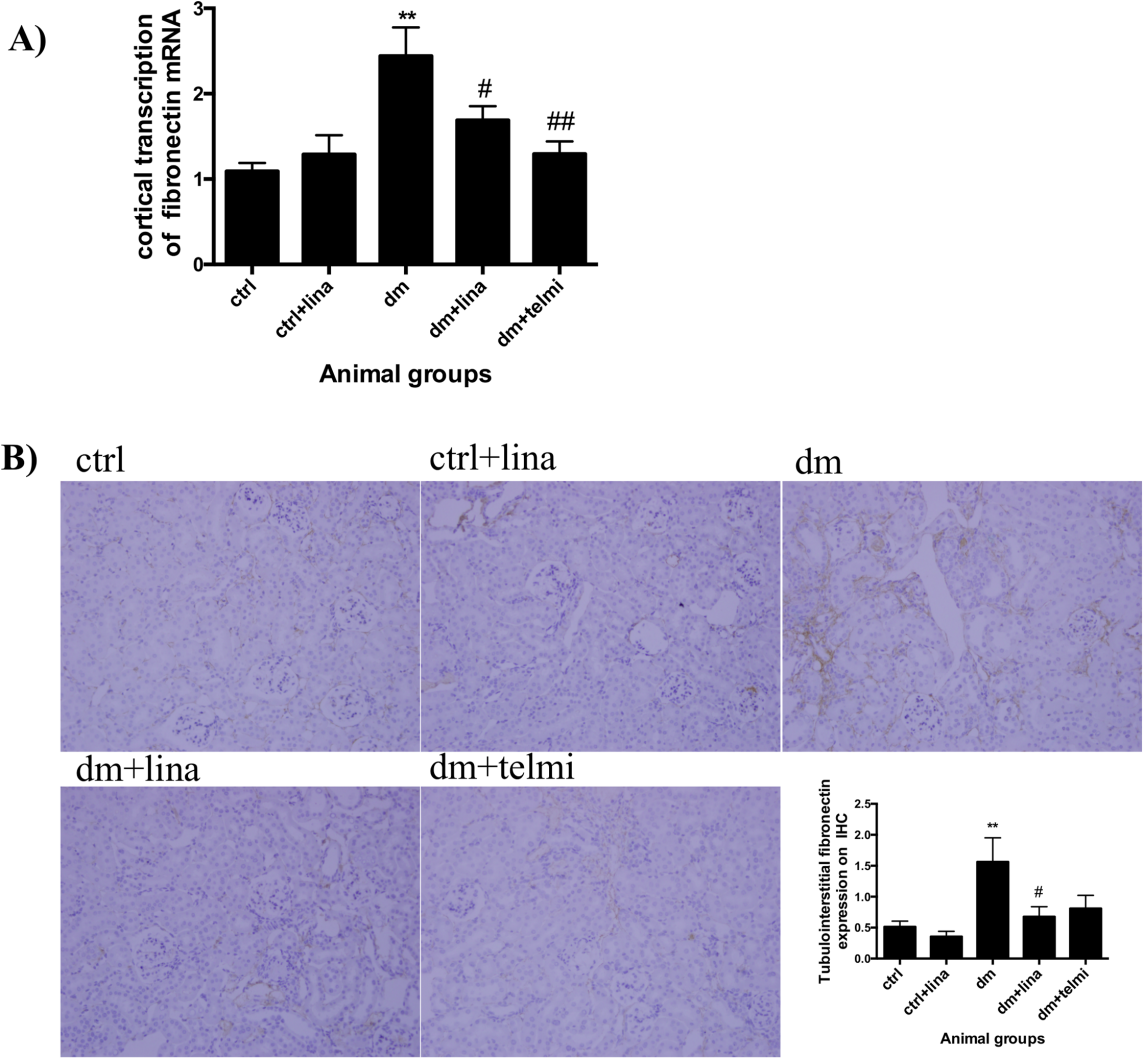
Linagliptin reduced fibronectin transcription and expression in diabetic mice. **(A)** Diabetic mice demonstrated increased cortical fibronectin mRNA transcription by real time PCR. This was significantly reduced by linagliptin and telmisartan. **(B)** There was a significant increase in tubulointerstitial FN expression measured by immunohistochemistry in the diabetic animals and this was reduced with linagliptin. Representative photographs for control (ctrl), control + linagliptin (ctrl + lina), diabetic (dm), diabetic + linagliptin (dm + lina) and diabetic + telmisartan (dm + tel) groups are shown (Magnification = original X 200). Data are expressed as mean ± SEM with ** = P<0.01 vs ctrl, # = P<0.05 vs dm, ## = P<0.01 vs dm.

Diabetic mice showed significantly increased renal cortical transcription of fibronectin mRNA in comparison to control mice (P<0.01). This was significantly improved in diabetic mice treated with linagliptin (P<0.05) and telmisartan (P<0.01) (Fig 5A). In keeping with this, there was also a significant increase in tubulointerstitial fibronectin expression in diabetic mice (P<0.01), which was significantly reduced with linagliptin (P<0.05) (Fig 5B).

### Linagliptin did not reduce tubulointerstitial collagen deposition in diabetic mice

**Fig 6:**
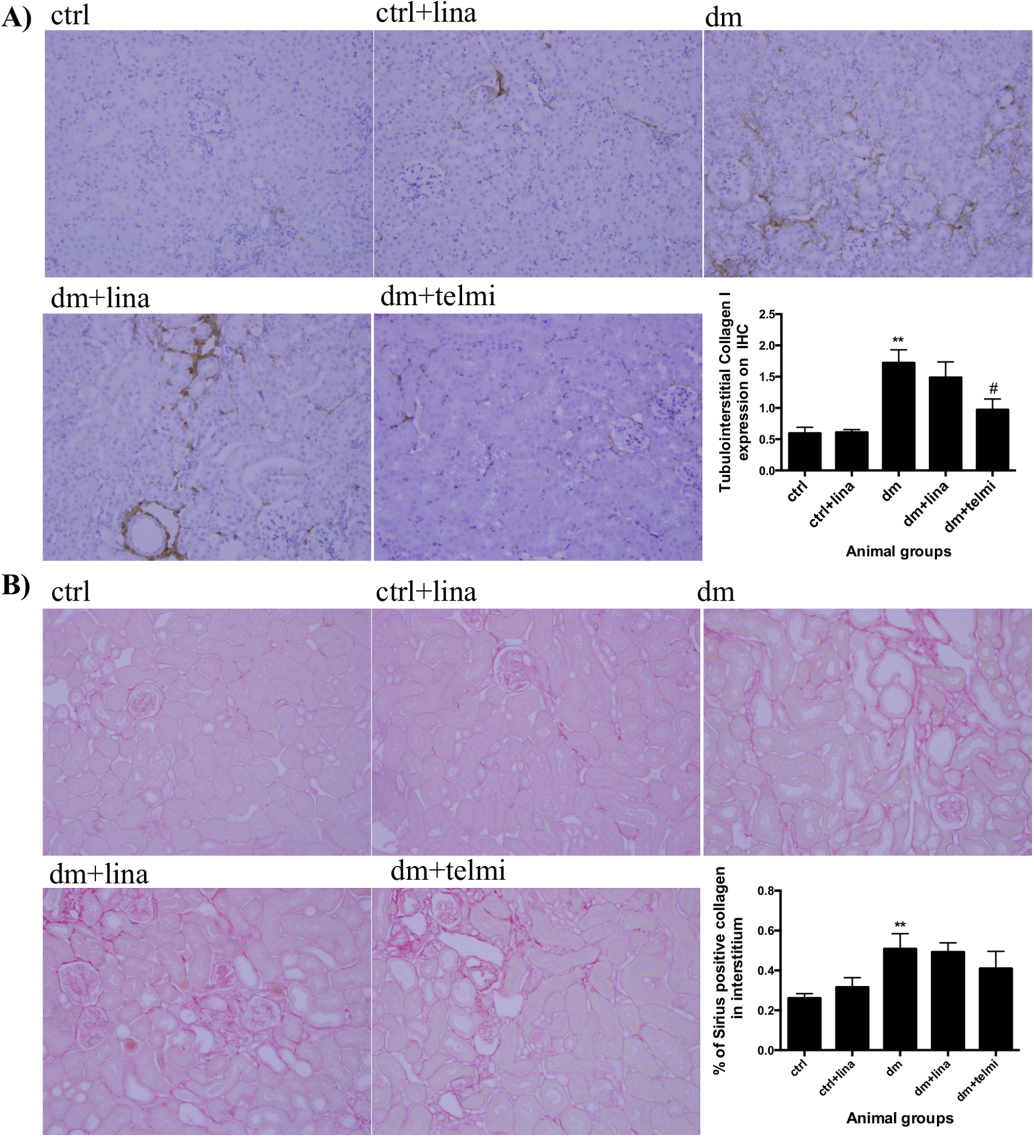
Linagliptin did not reduce tubulointerstitial collagen deposition in diabetic mice. **(A)** Diabetic mice showed increased collagen I and **(B)** picrosirius staining. Linagliptin did not change either. Telmisartan reduced collagen I expression. Representative photographs of collagen I and tubulointerstitial picrosirius red stain for control (ctrl), control + linagliptin (ctrl + lina), diabetic (dm), diabetic + linagliptin (dm + lina) and diabetic + telmisartan (dm + tel) groups are shown (Magnification = original X 200). Quantification was done using Image J. Data are expressed as mean ± SEM with ** = P<0.01 vs ctrl, # = P<0.05 vs dm.

Collagen I was increased in diabetic mice compared to control values (P<0.01). Telmisartan reduced collagen I deposition (P<0.05) (Fig 6A). Diabetic mice demonstrated an expected increase in renal picrosirius staining (collagen I and III) in comparison to control mice (P<0.01). Neither linagliptin nor telmisartan reduced picrosirius staining (Fig 6B).

## Discussion

This data provides new knowledge on the mechanism by which the DPP4 inhibitor, linagliptin, reduces active TGFß1 and downstream renal fibrotic markers. An important aspect of this novel finding is that the interaction between CIM6PR and DPP4 is “switched on” by high glucose, and hence is maximally modulated by linagliptin in this environment. In the presence of excess M6P, the CIM6PR binding sites become saturated, resulting in the reduction in CIM6PR/DPP4 interaction, which would suggest that the interaction is occurring through a M6P residue on the DPP4 molecule. The fact that linagliptin also reduced this interaction, suggests a M6P mediated mechanism which is independent of GLP-1 as our in vitro system is lacking in GLP-1. This finding is also confirmed in our *in vivo* model of diabetic nephropathy. Linagliptin was able to reduce renal cortical fibronectin transcription and tubular pSmad2/3 expression on immunohistochemistry. We also noted a trend towards reduction in pSmad2 expression on western blot analysis of whole kidney tissue. These findings are suggestive of inhibition of active TGFß1 signalling. There was also a concomitant trend towards a reduction in tubular atrophy. Importantly this anti-fibrotic trend was independent of glucose or blood pressure lowering.

The renal effects of DPP4 inhibition have been previously explored in animal models of Type 1 and Type 2 diabetes. Both studies that have looked at the effect of DPP4 inhibition (using vildagliptin and sitagliptin) show renoprotection. However the HbA1c in the DPP4 inhibitor treated diabetic animals was lower than in the diabetic control animals [9, 10]. So in both of these *in vivo* studies it is difficult to conclude that the renal effects of DPP4 inhibition are independent of glucose lowering. Kanasaki et al investigated the antifibrotic effect of linagliptin in a type 1 model of diabetic nephropathy. This study demonstrates that linagliptin after 4 weeks ameliorated diabetic kidney fibrosis independent of glucose lowering and this was in association with the inhibition of endothelial–mesenchymal transition and the restoration of microRNA29 a/b/c [11]. Like this study, the advantage of our experimental design is ensuring that the glucose levels are matched so the renal findings are not because of expected changes one would see with glucose lowering itself. In contrast to Kanasaki et al our study has a much longer duration of treatment (20 weeks) with linagliptin in the diabetic mice.

The potential advantage over renin-angiotensin blockade such as telmisartan or other anti-fibrotic therapies targeting TGFß1, such as monoclonal antibodies, is the finding that CIM6PR and DPP4 interaction seems to be occurring selectively in context of high glucose and hence maximally modulated by linagliptin during hyperglycaemia. Specifically targeting TGFß1 utilizing antibodies is challenging clinically because of the complexity of its biological role in various organs. Non-selective targeting of TGFß is unlikely to be a promising therapeutic strategy as TGFß1 knockout is a lethal phenotype [12] and drugs that non-specifically target TGFß1 including monoclonal antibodies result in cancer, inflammation and autoimmune disease. Hence more targeted therapies are required. One strategy to overcome this is to exploit the fact that different cells activate latent TGFß1 using different proteins. For example, unlike the kidney proximal tubular cells, the immune system regulates TGFß1 through integrins rather than CIM6PR [13], highlighting the potential for DPP4 inhibitors as antifibrotic in a high glucose environment being limited to CIM6PR dependent TGFß1activation. In terms of using M6P analogues directly to achieve the same effect as linagliptin, their use is currently limited by a short half-life, low bioavailability and poor receptor binding [14, 15].

Although our study suggests that linagliptin has anti-TGFß1 signalling effects in the kidney, we did not see any significant change in the glomerulosclerotic index or tubulointerstitial collagen deposition and albuminuria, which reflects the multifactorial aetiology of diabetic nephropathy. The polyol pathway is activated in cells such as the proximal tubular cells where glucose entry is independent of insulin. So, this finding is in keeping with our hypothesis and previous findings that DPP4 inhibition is more likely to alter extracellular mediators such as TGFß1 but not the intracellular regulation induced by hyperglycaemia [3]. Hence we would propose that effective treatments to limit diabetic nephropathy necessarily involve targeting multiple pathophysiological pathways, which include inhibition of membrane bound DPP4.

Our study has a few limitations. We have shown indirectly rather than directly how linagliptin influences the interaction between CIM6PR and DPP4. Linagliptin binds to an internalised site in the enzyme and removed from the M6P moiety on the DPP4 molecule and does not result in significant conformational change (communication from Boehringer-Ingelheim, Germany). So although our data supports a M6P related mechanism, this could be through inhibiting either an enzymatic or non-enzymatic property of DPP4. Similar findings were published by Ishibashi et al where blocking the interaction of DPP-4 with M6P/IGF-IIR by the addition of excess amount of free M6P or M6P/IGF-IIR-Ab completely inhibited the DPP-4-induced increase in superoxide generation in HUVECs[16]. It is also important to appreciate that whilst our *in vitro* data supports a non GLP-1 mediated mechanism, when linagliptin is administered *in vivo* it results in raised GLP-1 levels as expected. Hence our findings do not delineate between a GLP-1 and non GLP-1 mediated mechanism. However, this is mechanistically irrelevant when considering human application.

In summary, we propose novel mechanistic data that adds to the existing body of knowledge that DPP4 inhibition with linagliptin can inhibit the TGFß1 related fibrotic pathway in diabetic nephropathy. Clinical trials are in progress to determine whether linagliptin impacts on renal and cardiovascular (ClinicalTrials.gov Identifier: NCT01897532) outcomes. If this is also borne out in clinical trials, DPP4 inhibition with linagliptin may have an added advantage in those at risk of diabetic nephropathy.
